# Peer Review of “Google Trends as a Predictive Tool for COVID-19 Vaccinations in Italy: Retrospective Infodemiological Analysis”

**DOI:** 10.2196/38665

**Published:** 2022-04-19

**Authors:** Artur Strzelecki

**Affiliations:** 1 Department of Informatics University of Economics in Katowice Katowice Poland

**Keywords:** COVID-19, epidemiology, Google Trends, infodemiology, infoveillance, Italy, public health, SARS-CoV-2, vaccinations, vaccines, social media analysis, social media

*This is a peer-review report submitted for the paper “Google Trends as a Predictive Tool for COVID-19 Vaccinations in Italy: Retrospective Infodemiological Analysis.”*

## Round 1 Review

### General Comments

The subject of the brief paper [1] “Google Trends as a Predictive Tool for COVID-19 Vaccinations in Italy: a Retrospective Infodemiological Analysis” is timely and valuable to the audience of JMIRx Med. Overall, the paper is well structured, reads exceptionally well, and covers the existing literature quite well. The analysis of the data is interesting and well documented.

The author of the paper has selected keywords used in the Google Search engine, which could reveal an intention to take a vaccine against COVID-19 in Italy and compared this interest with headlines in the second most read newspaper in Italy. The paper has a transparent and replicable procedure to collect data and do statistical tests.

The results show a marked and significant cross-correlation between web queries on vaccine reservations and actual vaccinations against COVID-19 in Italy. On the other hand, the cross-correlation between vaccine-related news and vaccine web searches is low.

### Specific Comments

#### Minor Comments

1. I think that the limitations of this study are much broader than those listed in the work. There is a strong vaccine hesitation movement across different European countries, which could at least be mentioned in the work. The authors only noticed news in a newspaper on rare side effects of vaccination. This is what strongly influences, on the one hand, queries entered into a search engine and, on the other hand, a decrease in the number of vaccinations.
